# Determination of the Role and Active Sites of PKC-Delta-Like from Lamprey in Innate Immunity

**DOI:** 10.3390/ijms20133357

**Published:** 2019-07-09

**Authors:** Yang Xu, Huan Zhao, Yang Tian, Kaixia Ren, Nan Zheng, Qingwei Li

**Affiliations:** 1 College of Life Science, Liaoning Normal University, Dalian 116081, China; 2 Lamprey Research Center, Liaoning Normal University, Dalian 116081, China

**Keywords:** PKC-δ-like, LPS, innate immunity, activity, lamprey

## Abstract

Protein kinase C-δ (PKC-δ) is an important protein in the immune system of higher vertebrates. Lampreys, as the most primitive vertebrates, have a uniquevariable lymphocyte receptor (VLR) immune system. PKC-δ-like is a crucial functional gene in lampreys and is highly expressed in their immune organs. In this study, lampreys were stimulated with different immunogens, and lipopolysaccharide (LPS) was found to increase the expression of PKC-δ-like. Overexpression of PKC-δ-like could also effectively activate the innate immune response. We further demonstrated that PKC-δ-like-CF, a catalytic fragment of PKC-δ-like, is responsible for activating the innate immune response, and Thr-211, which is Thr-419 of PKC-δ-like, was confirmed to be the key site affecting PKC-δ-like-CF activity. These results indicated that PKC-δ-like from lamprey may have an important role in the innate immune response.

## 1. Introduction

Protein kinase C (PKC) was initially identified and characterized as a protein hydrolysis-activated kinase called protein kinase M [1,2]. It has been established that PKC is a family of at least 12 serine/threonine kinases that is divided into three subfamilies: The classical PKCs (α, β1, β2, and γ), which are activated by diacylglycerol (DAG) and calcium; the novel PKCs (δ, ε, η, and θ) which are activated by DAG; and the atypical PKCs (ζ and λ/ι), which respond to neither DAG nor calcium. The PKCs mentioned above play a central role in cell signaling pathways involving cell proliferation, differentiation, apoptosis, and the immune system. Protein kinase C-δ (PKC-δ) is the first identified member of the novel PKC subfamily. It was discovered in 1986, and some articles have reported its structure and function [3,4]. It is confirmed that PKC-δ is widely expressed, is activated by many exogenous stimuli, and is an important regulator of peripheral lymphocyte development and immune homeostasis [5,6]. Human PKC-δ plays an important role in the innate immune response [7,8,9,10,11,12].

Lampreys are considered to be the most ancient living vertebrates. They are jawless vertebrates and can be traced back to 530 million years ago [13,14,15]. Because of their unique evolutionary position between jawless and jawed vertebrates, lampreys are an important species for studying the evolution of the immune system [16]. Lampreys have a unique immune system; the variable lymphocyte receptors (VLRs) in lamprey function similarly to the immunoglobulin receptors in higher vertebrates, specifically recognizing and responding to external pathogens [17,18,19,20,21]. Many extant studies investigating lampreys have focused on their immune system. Therefore, the description of crucial genes in the innate immune system of lamprey are helpful for understanding the origin and evolution of the innate immune system.

In a previous study, we reported the identification of a PKC-δ homologue named PKC-δ-like in the lamprey *Lampetra japonica* (*L. japonica*) [22]. PKC-δ-like was detected only in the supraneural body, which is an important immune organ in lamprey [16,23]. The high expression level of PKC-δ-like in the supraneural body indicated that this protein might play a role in lamprey immunity. In this study, we investigated the role of PKC-δ-like from lamprey in the innate immunity.

## 2. Results

### 2.1. The Effect of Different Antigenic Stimuli on the Leukocytes of Lamprey

**Figure 1:**
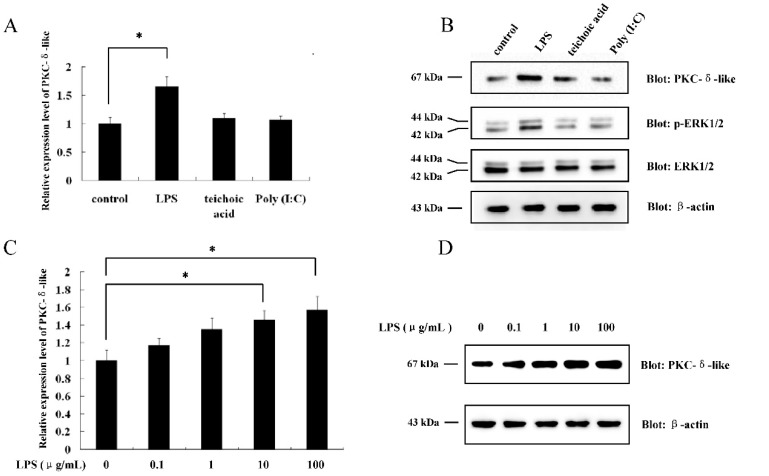

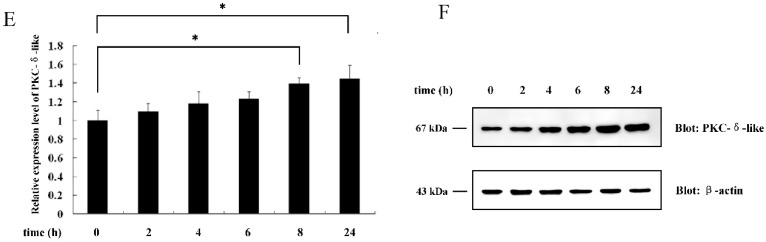
The effect of different immunogens on the expression of protein kinase C-δ-like (PKC-δ-like) in the leukocytes of *L. japonica*. (**A**) The lampreys were immunized withlipopolysaccharide(LPS), teichoic acid, Poly (I:C), and control. The mRNA levels of PKC-δ-like in the leukocytes of *L. japonica* were detected. (**B**) The lampreys were immunized with LPS, teichoic acid, Poly (I:C), and control. The protein levels of PKC-δ-like, together with the phosphorylation level of ERK1/2, in the leukocytes of *L. japonica* were detected. (**C**) The leukocytes of *L. japonica* were incubated with LPS at different concentrations (0, 0.1, 1, 10, 100 μg/mL). The mRNA levels of PKC-δ-like were detected. (**D**) The leukocytes of *L. japonica* were incubated with LPS at different concentrations (0, 0.1, 1, 10, 100 μg/mL). The protein levels of PKC-δ-like were detected. (**E**) The leukocytes of *L. japonica* were incubated with LPS for different hours (0, 2, 4, 6, 8, and 24 h). The mRNA levels of PKC-δ-like were detected. (**F**) The leukocytes of *L. japonica* were incubated with LPS for different hours (0, 2, 4, 6, 8, and 24 h). The protein levels of PKC-δ-like were detected. The asterisk indicates *p* < 0.05.

In our previous study, we found that PKC-δ-like had a high level of expression in the supraneural body of *L. japonica* [22]. It is known that leukocytes participate in the immune response of lamprey [24,25]. Thus, we determined whether PKC-δ-like was also expressed in the leukocytes of *L. japonica*. The results showed that the PKC-δ-like protein could be detected in the leukocytes (Figure S1). The transcriptional profile of PKC-δ-like was examined with a real-time quantitative PCR detection system (qPCR) in leukocytes after the lampreys were immunized with lipopolysaccharide (LPS), teichoic acid, and poly (I:C), as described in the Methods section. It was found that only LPS could increase the mRNA level of PKC-δ-like (Figure 1A). Then, we quantified the protein expression of PKC-δ-like under the stimulation of these different antigens and found that only LPS could enhance the protein expression of PKC-δ-like (Figure 1B). Meanwhile, phosphorylation level of ERK1/2, the downstream of the PKC pathway, was detected (Figure 1B). The results indicated that LPS also activated ERK1/2. Additionally, leukocytes were isolated from the blood of *L. japonica*, cultured in 1640 medium, and treated with LPS at different concentrations (0, 0.1, 1, 10, 100 μg/mL) for 8 h. The mRNA and protein levels of PKC-δ-like were detected by qPCR and Western blot, respectively. The data showed that both the mRNA and protein expression levels increased with increasing LPS concentration (Figure 1C,D). Next, the leukocytes were treated with 10 μg/mL LPS for 0, 2, 4, 6, 8, and 24 h, and the mRNA and protein levels of PKC-δ-like were quantified by qPCR and Western blot, respectively. Both the transcription and the protein expression levels of PKC-δ-like increased in a time-dependent manner, as shown in Figure 1E,F. The results above indicated that LPS was able to stimulate the mRNA and protein expression of PKC-δ-like in a concentration- and time-dependent manner.

### 2.2. Overexpression of PKC-δ-LikeCould Induce Cellular Inflammation 

**Figure 2:**
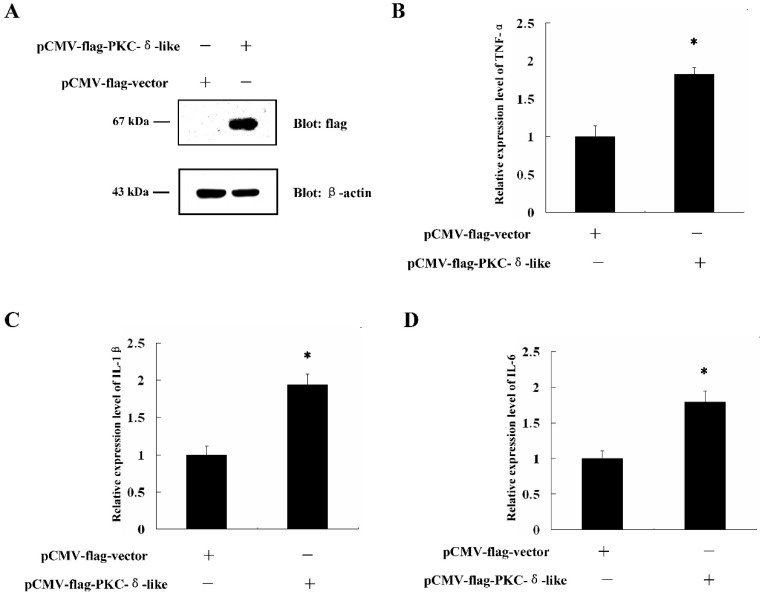
The effect of over-expression of PKC-δ-like on the cellular inflammation reaction. (**A**) The protein expression of PKC-δ-like in the RAW264.7 cells was detected. (**B**) The mRNA levels of TNF-α in the RAW264.7 cells, which were transfected with PKC-δ-like, were detected. (**C**) The mRNA levels of IL-1β in the RAW264.7 cells, which were transfected with PKC-δ-like, were detected. (**D**) The mRNA levels of IL-6 in the RAW264.7 cells, which were transfected with PKC-δ-like, were detected. The asterisk indicates *p* < 0.05.

It is known that when cells are treated with LPS, cellular inflammation is induced. As LPS enhances the expression of PKC-δ-like, we wanted to determine whether PKC-δ-like participates in cellular inflammation. Full-length, PKC-δ-like cDNA was cloned into the pCMV-flag vector and transfected into RAW264.7 cells (Figure 2A). The mRNA levels of TNF-α, IL-1β, and IL-6 were quantified with real-time PCR. The results showed that when the cells overexpressed PKC-δ-like, the levels of TNF-α, IL-1β, and IL-6 in cells were all increased (Figure 2B–D). The expression of TNF-α, IL-1β, and IL-6 is involved in the innate immune response. Thus, the results indicated that the overexpression of PKC-δ-like could induce cellular inflammation. 

### 2.3. The Catalytic Fragment of PKC-δ-Like Could also Stimulate Cellular Inflammation

**Figure 3:**
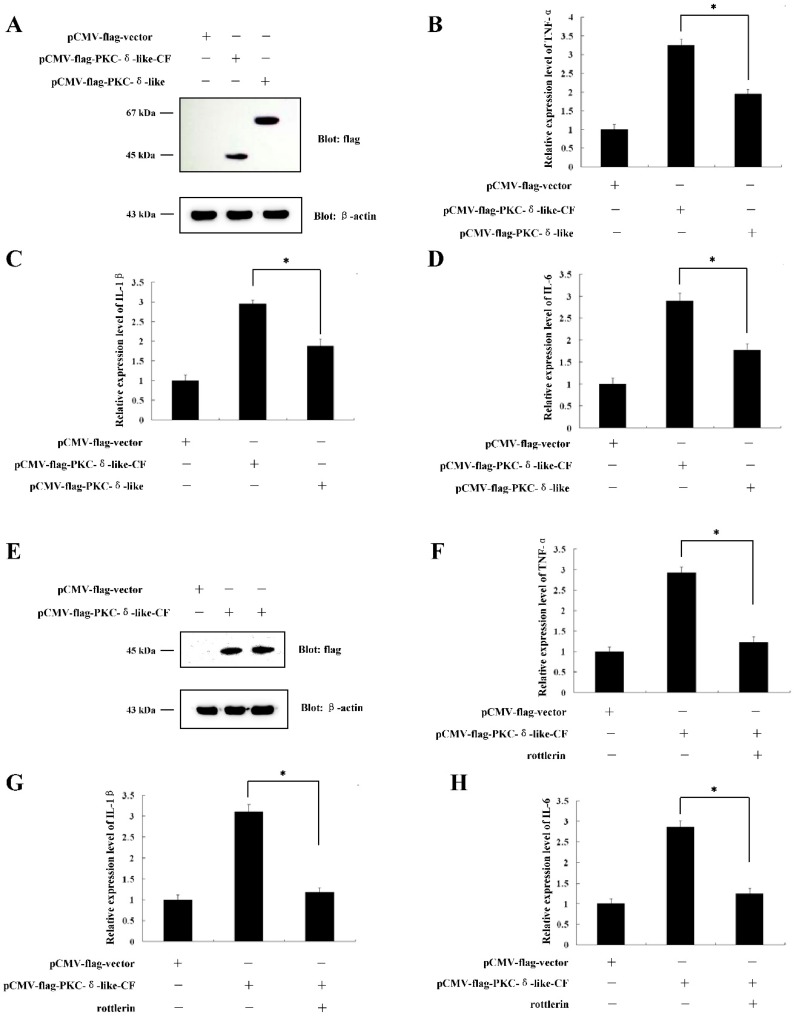
The effect of over-expression of PKC-δ-like-CF on the cellular inflammation reaction. (**A**) The protein expression of PKC-δ-like-CF and PKC-δ-like in the RAW264.7 cells was detected. (**B**) The mRNA levels of TNF-α in the RAW264.7 cells, which were transfected with PKC-δ-like-CF or PKC-δ-like, were detected. (**C**) The mRNA levels of IL-1β in the RAW264.7 cells, which were transfected with PKC-δ-like-CF or PKC-δ-like, were detected. (**D**) The mRNA levels of IL-6 in the RAW264.7 cells, which were transfected with PKC-δ-like-CF or PKC-δ-like, were detected. (**E**) The protein expression of PKC-δ-like-CF in the RAW264.7 cells was detected. (**F**) The mRNA levels of TNF-α in the PKC-δ-like-CF-transfected RAW264.7 cells, which were treated with or without rottlerin, were detected. (**G**) The mRNA levels of IL-1β in the PKC-δ-like-CF-transfected RAW264.7 cells, which were treated with or without rottlerin, were detected. (**H**) The mRNA levels of IL-6 in the PKC-δ-like-CF-transfected RAW264.7 cells, which were treated with or without rottlerin, were detected. The asterisk indicates *p* < 0.05.

PKC-δ-like has two domains, as described in our previous paper: The regulatory domain and the catalytic domain [22]. The catalytic domain (209 aa–589 aa) is inhibited by the regulatory domain when PKC-δ-like is inactivated. If the regulatory domain is removed, the catalytic fragment will be continuously activated. We added the catalytic domain to the pCMV-flag vector, and transfected the vector into RAW264.7 cells (Figure 3A). We sought to determine whether the catalytic fragment of PKC-δ-like (PKC-δ-like-CF) could also induce cellular inflammation. The results showed that PKC-δ-like-CF could significantly increase the mRNA levels of TNF-α, IL-1β, and IL-6 (Figure 3B–D). However, when we treated the cells that were transfected with the pCMV-flag-PKC-δ-like-CF (Figure 3E) with 10 μmol/L of rottlerin, an inhibitor of PKC-δ [26], the mRNA levels of TNF-α, IL-1β, and IL-6 decreased (Figure 3F–H). We did the activity assay of PKC-δ-like-CF with or without rottlerin to see whether rottlerin could inhibit the kinase activity of PKC-δ-like-CF. The results showed that rottlerin could reduce the activity of PKC-δ-like-CF in vitro (Figure S2). These results suggested that the inhibition of PKC-δ-like-CF activity could prevent PKC-δ-like-CF from inducing cellular inflammation.

### 2.4. Determination of PKC-δ-Like Active Sites of the Cellular Inflammation Reaction

**Figure 4:**
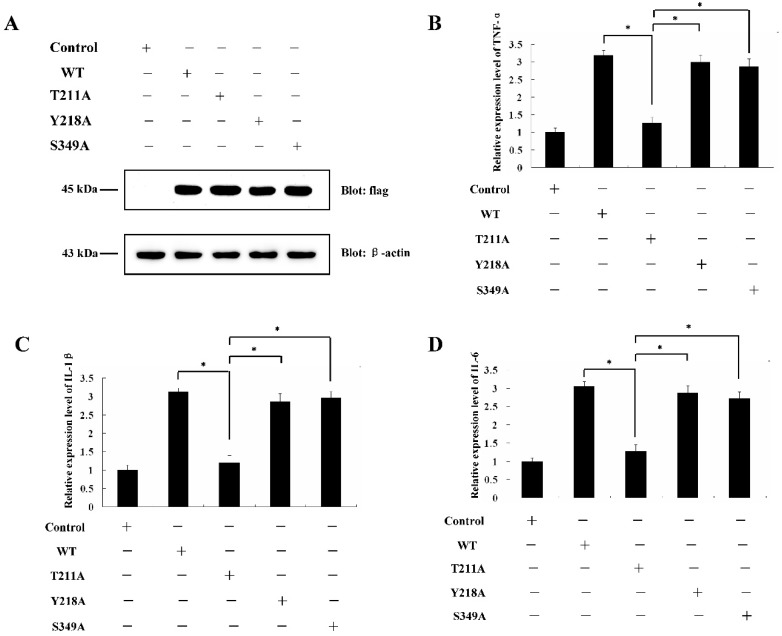
Determination of PKC-δ-like active sites on the cellular inflammation reaction. (**A**) The protein expression of WT, T211A, Y218A, or S349A in the RAW264.7 cells was detected. (**B**) The mRNA levels of TNF-α in the RAW264.7 cells, which were transfected with WT, T211A, Y218A, or S349A, were detected. (**C**) The mRNA levels of IL-1β in the RAW264.7 cells, which were transfected with WT, T211A, Y218A, or S349A, were detected. (**D**) The mRNA levels of IL-6 in the RAW264.7 cells, which were transfected with WT, T211A, Y218A, or S349A, were detected. (WT: Wild type of PKC-δ-like-CF; T211A: PKC-δ-like-CF (T211A); Y218A: PKC-δ-like-CF (Y218A); S349A: PKC-δ-like-CF (S349A).) The asterisk indicates *p* < 0.05.

**Figure 5:**
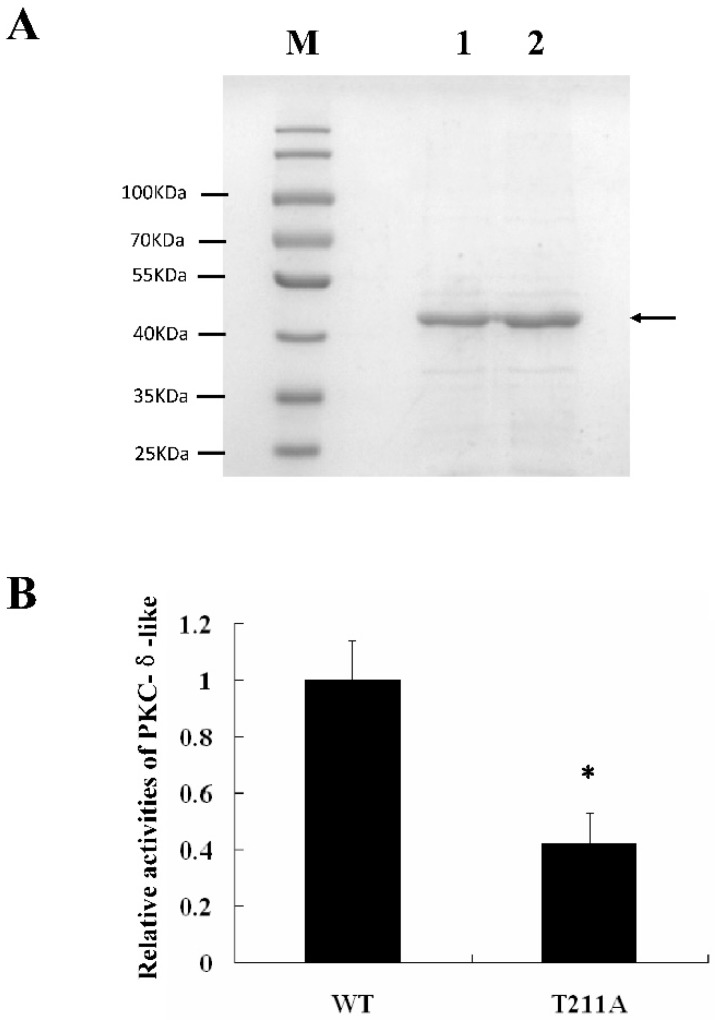
Activity assay of PKC-δ-like-CF and PKC-δ-like-CF (T211A) in vitro. (**A**) Expression and purification of PKC-δ-like-CF and PKC-δ-like-CF (T211A) recombinant protein. The recombinant proteins were expressed in *E. coli* BL21 and analyzed through SDS-PAGE. M, protein marker; lane 1, purified PKC-δ-like-CF recombinant protein; lane 2, purified PKC-δ-like-CF (T211A) recombinant protein. (**B**) Activity assay of PKC-δ-like-CF and PKC-δ-like-CF (T211A). The asterisk indicates *p* < 0.05.

It was reported that Thr-507, Tyr-514, and Ser-645 were all involved in the activation of PKC-δ in humans [27,28,29]. Sequence alignments of human PKC-δ protein and lamprey PKC-δ-like protein showed that Thr-419, Tyr-426, and Ser-557 might be the active site of PKC-δ-like (Figure S3). To confirm the exact active sites involved in the immune function of PKC-δ-like, we mutated Thr-211, Tyr-218, and Ser-349 in PKC-δ-like-CF, generating T211A, Y218A, and S349A, respectively. These three mutants were transfected into RAW264.7 cells (Figure 4A). The data showed that only the Thr-211 mutation impacted the ability of PKC-δ-like-CF to activate the immune response (Figure 4B–D). Both PKC-δ-like-CF (T211A) and PKC-δ-like-CF were constructed into the pET-28a vector and expressed in *Escherichia coli* BL21 (DE3). The proteins were both purified, and their activities were tested by using a PKC kinase activity kit (Enzo Life Sciences, Farmingdale, NY, U.S.A.) in vitro. As shown in Figure 5B, the activity of PKC-δ-like-CF (T211A) was 0.4-fold that of PKC-δ-like-CF. Figure 5A shows the expression of these two proteins by SDS-PAGE. 

## 3. Discussion

Lampreys not only have the most primitive organ and tissue morphology and structure but also differ greatly from higher vertebrates in terms of molecular heredity, protein function, and cellular signaling pathways [30,31]. In 2004, Pancer et al. published an article in Nature describing the differences in the adaptive immune systems of lampreys and higher vertebrates. Random combinations of “leucine-rich repeats” (LRRs) form a variety of VLRs, which can recognize foreign antigens, similar to antibodies in the adaptive immune system of higher vertebrates [32].

PKC-δ-like is an important functional gene identified in previous studies of *L. japonica* [22]. Moreover, we found that PKC-δ-like was highly expressed in the supraneural body, which is one of the most important immune organs of lampreys. Studies have shown that human PKC-δ plays an important role in the human immune system [33,34,35]. Studies have also shown that members of the human PKC family play different roles in immunity; for example, PKC-θ plays an important role in the maturation and activation of T lymphocytes, and PKC-δ functions in the activation of B lymphocytes and innate immunity [36,37,38]. However, current studies have shown that lampreys have not evolved an adaptive immune system similar to those of higher vertebrates. Therefore, we investigated whether PKC-δ-like plays a crucial role in the innate immunity of the lamprey. We first immunized *L. japonica* with representative immunogens of different pathogens: LPS from gram-negative bacteria, teichoic acid from gram-positive bacteria, and poly (I:C). Among these molecules, only LPS could increase the expression of PKC-δ-like in *L. japonica* leukocytes. In human cells, LPS activates the TLR-4-mediated signaling pathway of the innate immune system, teichoic acid activates TLR-2, and poly (I:C) activates TLR-3. Previous studies showed that human PKC-δ participates in innate immunity mainly through the TLR-4 pathway [38,39]. Therefore, we hypothesized that PKC-δ-like might be a member of the TLR-4 signaling pathway in lampreys. However, the genes that code the TLR-4 receptor and related proteins in the TLR-4 pathway of lampreys are still unknown, warranting further studies.

Studies have shown that PKC-δ is involved in the production of cytokines in the innate immune response [40,41,42,43]. To determine the exact role of PKC-δ-like in innate immunity, we overexpressed PKC-δ-like in RAW 264.7 cells and detected the expression of cytokines related to innate immunity. The results showed that overexpression of PKC-δ-like could activate the innate immune system of RAW 264.7 cells. Human PKC-δ has two domains, the regulatory domain at the N-terminal and the catalytic domain at the C-terminal. In the resting state, the regulatory domain is bound by the catalytic domain, and its activity is inhibited. When PKC-δ is stimulated by upstream signals, its conformation changes, and the catalytic domain is separated from the regulatory domain, thus liberating the catalytic domain and enabling it to be activated. Therefore, using molecular cloning, we removed the regulatory domain of PKC-δ-like and only expressed its catalytic domain, thus PKC-δ-like-CF was activated. We further overexpressed PKC-δ-like-CF in RAW 264.7 cells. The results showed that PKC-δ-like-CF was also able to activate the innate immune response, and that the activation effect of the catalytic fragment was greater than that of the full-length protein. This further indicated that the catalytic fragment of PKC-δ-like is likely the functional component of PKC-δ-like that participates in the innate immune response.

To further confirm the amino acid sites in PKC-δ-like-CF that play a key role in its activity, we compared the amino acid sequences of human PKC-δ and lamprey PKC-δ-like. The results of the sequence alignment suggested that three amino acid sites, Thr-419, Tyr-426, and Ser-557, of lamprey PKC-δ-like were homologous to the three human PKC-δ amino acid sites, Thr-507, Tyr-514, and Ser-645, known to be responsible for PKC-δ activity. Therefore, we mutated the corresponding Thr-211, Tyr-218, and Ser-349 amino acid sites in PKC-δ-like-CF into inactive Ala residues. Subsequently, the effects of overexpression of the three mutants on the activation of cellular innate immunity were examined. The results showed that PKC-δ-like-CF (T211A) could not effectively stimulate cells to induce the innate immune response. This further indicated that the Thr-419 amino acid is necessary for PKC-δ-like to participate in the innate immune response. Additionally, the in vitro detection of kinase activity indicated that the mutated amino acid site influenced the kinase activity of PKC-δ-like. Although we have demonstrated that PKC-δ-like plays an important role in the innate immune response of the lamprey, and we identified its active domain and key amino acid sites, the immune-related signaling pathway in which PKC-δ-like is involved, and its upstream and downstream interacting proteins, require further research and confirmation. Because lampreys are the most primitive vertebrates, their innate immune system is quite different from that of higher vertebrates. Meanwhile, the gene information of many related proteins in the TLR-4 pathway is unknown because the genetic information of lampreys is not as complete as that of other common model animals, which hinders the determination of the exact location and mechanism of PKC-δ-like in this pathway. Therefore, we could only preliminarily explore the role of PKC-δ-like in innate immunity, but the specific mechanism is not clear. The number and nature of the specific members of the human PKC family are known, but the genetic information of other members of the PKC family of lampreys is unknown, which increases the difficulty of systematically studying the role of the entire PKC family in innate immunity. In summary, additional work is needed to confirm the exact mechanism of PKC-δ-like in the innate immune response of lampreys.

## 4. Material and Methods

### 4.1. Animals

Fresh adult male *L. japonica* (length: 36.4–58.4 cm, weight: 112–274.5 g) were obtained from Tongjiang River Basin of Songhua River in Heilongjiang Province in December 2017. The work was approved by the Institute of Animal Welfare and the Ethics Committee of Dalian Medical University (License No. SYXK2004-0029), and carried out in accordance with the approved guidelines. The animals were divided into four groups (10 animals per group). One group of animals were immunized with LPSsolution, carried out by injecting the lamprey intraperitoneally with 0.1 mg of LPS in 0.1 mL 0.9% sodium chloride for each animal. One group of animals were immunized with teichoic solution, carried out by injecting the lamprey intraperitoneally with 0.1 mg of teichoic in 0.1 mL 0.9% sodium chloride for each animal. One group of animals were immunized with Poly (I:C) solution, carried out by injecting the lamprey intraperitoneally with 0.1 mg of Poly (I:C) in 0.1 mL 0.9% sodium chloride for each animal. The control group was injected with normal saline. Animals were immunized at 7-day intervals by three injections. On the third day after the last immunization, the animals were killed for tissue samples.

### 4.2. Isolation of Leukocytes and Cell Culture

*L. japonica* peripheral blood was collected from the caudal subcutaneous sinus. Leukocytes were enriched with Ficoll-Paque medium (concentration: 1.092 g/mL) by Ficoll-Paque gradient centrifugation. After centrifugation, leukocytes were collected and cultured in RPMI 1640 medium containing 10% fetal bovine serum. All cell cultures were cultured at 37 °C in a 5% CO_2_ atmosphere.

### 4.3. Real-Time PCR

The expression of PKC-δ-like, TNF-α, IL-1β, or IL-6 messenger RNA (mRNA) was detected by a real-time quantitative PCR detection system (qPCR). Total RNA was isolated from lamprey tissues or cells using RNAiso reagent (TaKaRa Biotechnology, Dalian, China). Total RNA was treated with DNase I (TaKaRa Biotechnology, Dalian, China) and then reverse transcription was performed using PrimeScript^TM^ RT kit (Perfect Real Time) (TaKaRa Biotechnology, Dalian, China). qPCR experiments were performed with a TaKaRa TP800 real-time PCR system (TaKaRa Biotechnology, Dalian, China), using 2 μL cDNA and 16.8 μL SYBR green PCR mastermix (TaKaRa Biotechnology, Dalian, China), and 0.6 μL of each specific primer as shown in Table 1. Glyceraldehyde-3-phosphate dehydrogenase (GAPDH) of lampreys or mice was used as an internal control to standardize the starting amount of RNA. The results are expressed as the mean ± SD of three parallel experiments.

### 4.4. Western Blotting

The tissue or cells are lysed in the cell lysis buffer for Western and IP (Beyotime, Shanghai, China) and phenylmethanesulphonyl fluoride (PMSF) (Beyotime, Shanghai, China). The lysate was centrifuged at 12,000× *g* for 20 min at 4 °C, then 80 μL of supernatant was added to 20 μL of 5×loading buffer and boiled at 100 °C for 10 min. The proteins were analyzed by SDS-PAGE, transferred to a polyvinylidene fluoride (PVDF) membrane (Millipore Corporation, Billerica, MA, USA), and blocked with 5% skim milk containing 0.05% Tween (TBS-T). After washing three times in TBS-T, the membrane was incubated with anti-PKC-δ-like antibody (1 μg/mL) or anti-flag antibody (Abcam, Cambridge, UK) (1 μg/mL) at 4 °C TBS-T and 5% skim milk overnight. After washing, the membrane was incubated with goat anti-rabbit IgG conjugated with horseradish peroxidase (HRP) in TBS-T and 5% skim milk at 25 °C for 1 h. Signals were displayed using Enhanced Chemiluminescence (ECL) kits (Sangon Biotech, Shanghai, China). Rabbit anti-β-actin (Sangon Biotech, Shanghai, China) is used for standardized protein quality in each lane.

### 4.5. Cell Culture and Transfection

RAW264.7 mouse macrophages were cultured in DMEM (Invitrogen Co., Grand Island, NY, USA) supplemented with 10% FBS, 1% penicillin/streptomycin (Invitrogen Co., Grand Island, NY, USA), and 4 mM L-glutamine at 37 °C in a 5% CO_2_ atmosphere. The transfection of the plasmids into the cells was performed using FuGENE™ HD Transfection Reagent (Promega, WI, USA) according to the manufacturer’s instructions. 

### 4.6. Construction of PKC-δ-Like-CF Mutants

Mutant forms of T211A, Y218A, and S349A were prepared using QuikChange II-E Site-Directed Mutagenesis Kit (Agilent Technologies, Santa Clara, CA, USA). Plasmids with wild-type genes were used as templates and primers (Table 1) were designed according to the manufacturer’s manual. The mutant plasmids were sequenced by the company (Sangon Biotech, Shanghai, China).

### 4.7. PKC-δ Kinase Activity Assay

The activities of PKC-δ-like-CF and PKC-δ-like-CF (T211A) were measured using a PKC kinase activity kit (Enzo Life Sciences, Farmingdale, NY, USA) according to the manufacturer’s instructions. Briefly, the purified protein was added with 10 μL ATP. They were incubated for up to 90 min at 30 °C. Then, 40 μL of Phosphospecific Substrate Antibody was added and incubated at room temperature for 60 min. Then, 40 μL of diluted Anti-Rabbit IgG:HRP Conjugate was added and incubated at room temperature for 30 min. Then, 60 μL of TMB Substrate was added and incubated at room temperature for 30–60 mis. Incubation time should be determined by the investigator according to color development. Then, 20 μL of Stop Solution 2 was added to each reaction system. Finally, the absorbance at 450 nm was measured by SpectraMax i3x (Molecular Devices, CA, USA). 

### 4.8. Statistical Analysis

Data were presented as mean ± SD of at least three experiments. The differences between different groups were analyzed using student’s *t*-test and analysis of variance (ANOVA). *p* < 0.05 was considered statistically significant.

## Figures and Tables

**Table 1:** Oligonucleotide Primers Used in the Study.

Primer	Sequence (5′-3′)
***Realtime-PCR***	
PKC-δ-like-forward	GCATCTCCACGGAACGAC
PKC-δ-like-reverse	CCACCTCCACCTTCTCAACT
Lamprey-GAPDH-forward	ACCCCTTCATTGACCTGGAGTA
Lamprey-GAPDH-reverse	TGCTTACCCCATGGGATGTT
TNF-α-forward	GTCTCAGCCTCTTCTCATTC
TNF-α-reverse	CATAGAACTGATGAGAGGGA
IL-1β-forward	AAATACCTGTGGCCTTGGGC
IL-1β-reverse	CTTGGGATCCACACTCTCCAG
IL-6-forward	GAGTCCTTCAGAGAGATACAG
IL-6-reverse	CTGTGACTCCAGCTTATCTG
human-GAPDH-forward	TGGCCAAGGTCATCCATGACAAC
human-GAPDH-reverse	TCCAGAGGGGCCATCCACAGTCTTCTG
***Mutation***	
F-T211A	AGGAAACCTCGCCACCGCGTTCTGCGGCACCCCGG
R-T211A	CCGGGGTGCCGCAGAACGCGGTGGCGAGGTTTCCT
F-Y218A	CTGCGGCACCCCGGACGCCATCGCACCCGAGATCT
R-Y218A	AGATCTCGGGTGCGATGGCGTCCGGGGTGCCGCAG
F-S349A	CGAGTCCCCGCAGGTGGCGTGCCGAGGAAACAGCA
R-S349A	TGCTGTTTCCTCGGCACGCCACCTGCGGGGACTCG

## Supplementary Materials

Supplementary materials can be found at https://www.mdpi.com/1422-0067/20/13/3357/s1.

## Abbreviations

PKC	Protein kinase C
*L. japonica*	*Lampetra japonica*
VLR	variable lymphocyte receptor
LPS	lipopolysaccharide
PKC-δ-like-CF	catalytic fragment of PKC-δ-like
WT	wild type of PKC-δ-like-CF
LRR	leucine-rich repeat
mRNA	messenger RNA
qPCR	real-time quantitative PCR
GAPDH	Glyceraldehyde-3-phosphate dehydrogenase
PMSF	phenylmethanesulphonyl fluoride
PVDF	polyvinylidene fluoride
ECL	Enhanced Chemiluminescence

## References

[1] Inoue M., Kishimoto A., Takai Y., Nishizuka Y. (1977). Studies on a cyclic nucleotide-independent protein kinase and its proenzyme in mammalian tissues. II. Proenzyme and its activation by calcium-dependent protease from rat brain. J. Biol. Chem..

[2] Takai Y., Kishimoto A., Inoue M., Nishizuka Y. (1977). Studies on a cyclic nucleotide-independent protein kinase and its proenzyme in mammalian tissues. I. Purification and characterization of an active enzyme from bovine cerebellum. J. Biol. Chem..

[3] Ono Y., Fujii T., Ogita K., Kikkawa U., Igarashi K., Nishizuka Y. (1987). Identification of three additional members of rat protein kinase C family: Delta-, epsilon- and zeta-subspecies. FEBS Lett..

[4] Gschwendt M., Kittstein W., Marks F. (1986). A novel type of phorbol ester-dependent protein phosphorylation in the particulate fraction of mouse epidermis. Biochem. Biophys. Res. Commun..

[5] Mecklenbräuker I., Saijo K., Zheng N.Y., Leitges M., Tarakhovsky A. (2002). Protein kinase Cdelta controls self-antigen-induced B-cell tolerance. Nature.

[6] Miyamoto A., Nakayama K., Imaki H., Hirose S., Jiang Y., Abe M., Tsukiyama T., Nagahama H., Ohno S., Hatakeyama S. (2002). Increased proliferation of B cells and auto-immunity in mice lacking protein kinase Cdelta. Nature.

[7] Blake R.A., Garcia-Paramio P., Parker P.J., Courtneidge S.A. (1999). Src promotes PKCdelta degradation. Cell Growth Differ..

[8] Cho W. (2001). Membrane targeting by C1 and C2 domains. J. Biol. Chem..

[9] Konishi H., Tanaka M., Takemura Y., Matsuzaki H., Ono Y., Kikkawa U., Nishizuka Y. (1997). Activation of protein kinase C by tyrosine phosphorylation in response to H_2_O_2_. Proc. Natl. Acad. Sci. USA.

[10] Kronfeld I., Kazimirsky G., Lorenzo P.S., Garfield S.H., Blumberg P.M., Brodie C. (2000). Phosphorylation of protein kinase C delta on distinct tyrosine residues regulates specific cellular functions. J. Biol. Chem..

[11] Kumar V., Pandey P., Sabatini D., Kumar M., Majumder P.K., Bharti A., Carmichael G., Kufe D., Kharbanda S. (2000). Functional interaction between RAFT1/FRAP/mTOR and protein kinase cdelta in the regulation of cap-dependent initiation of translation. EMBO J..

[12] Pappa H., Murray-Rust J., Dekker L.V., Parker P.J., McDonald N.Q. (1998). Crystal structure of the C2 domain from protein kinase C-delta. Structure.

[13] Osório J., Rétaux S. (2008). The lamprey in evolutionary studies. Dev. Genes Evol..

[14] Kuratani S., Kuraku S., Murakami Y. (2002). Lamprey as an evo-devo model: Lessons from comparative embryology and molecular phylogenetics. Genesis.

[15] Nikitina N., Bronner-Fraser M., Sauka-Spengler T. (2009). The sea lamprey *Petromyzon marinus*: A model for evolutionary and developmental biology. Cold Spring Harb. Protoc..

[16] Amemiya C.T., Saha N.R., Zapata A. (2007). Evolution and development of immunological structures in the lamprey. Curr. Opin. Immunol..

[17] Herrin B.R., Cooper M.D. (2010). Alternative adaptive immunity in jawless vertebrates. J. Immunol..

[18] Pancer Z., Cooper M.D. (2006). The evolution of adaptive immunity. Annu. Rev. Immunol..

[19] Hirano M., Das S., Guo P., Cooper M.D. (2011). The evolution of adaptive immunity in vertebrates. Adv. Immunol..

[20] Boehm T., McCurley N., Sutoh Y., Schorpp M., Kasahara M., Cooper M.D. (2012). VLR-based adaptive immunity. Annu. Rev. Immunol..

[21] Cooper M.D., Alder M.N. (2006). The evolution of adaptive immune systems. Cell.

[22] Xu Y., Zhu S., Zhao H., Li Q. (2017). Identification and characterisation of lamprey protein kinase C delta-like gene. Sci. Rep..

[23] Pang Y., Li C., Wang S., Ba W., Yu T., Pei G., Bi D., Liang H., Pan X., Zhu T. (2017). A novel protein derived from lamprey supraneural body tissue with efficient cytocidal actions against tumor cells. Cell Commun. Signal..

[24] Su P., Liu X., Han Y., Zheng Z., Liu G., Li J., Li Q. (2013). Identification and characterization of a novel IκB-ε-like gene from lamprey (*Lampetra japonica*) with a role in immune response. Fish. Shellfish Immunol..

[25] Su P., Liu X., Pang Y., Liu C., Li R., Zhang Q., Liang H., Wang H., Li Q. (2017). The archaic roles of the lamprey NF-κB (lj-NF-κB) in innate immune responses. Mol. Immunol..

[26] Ahn B.K., Jeong S.K., Kim H.S., Choi K.J., Seo J.T., Choi E.H., Ahn S.K., Lee S.H. (2006). Rottlerin, a specific inhibitor of protein kinase C-delta, impedes barrier repair response by increasing intracellular free calcium. J. Investig. Dermatol..

[27] Basu A. (2003). Involvement of protein kinase C-delta in DNA damage-induced apoptosis. J. Cell Mol. Med..

[28] Gschwendt M. (1999). Protein kinase C delta. Eur. J. Biochem..

[29] Zhao M., Xia L., Chen G.Q. (2012). Protein kinase c δ in apoptosis: A brief overview. Arch. Immunol. Ther. Exp. (Warsz.).

[30] Huxley T.H. (1876). The Nature of the Craniofacial Apparatus of Petromyzon. J. Anat. Physiol..

[31] Xu Y., Zhu S.W., Li Q.W. (2016). Lamprey: A model for vertebrate evolutionary research. Zool. Res..

[32] Pancer Z., Amemiya C.T., Ehrhardt G.R., Ceitlin J., Gartland G.L., Cooper M.D. (2004). Somatic diversification of variable lymphocyte receptors in the agnathan sea lamprey. Nature.

[33] Sun X., Wu F., Datta R., Kharbanda S., Kufe D. (2000). Interaction between protein kinase C delta and the c-Abl tyrosine kinase in the cellular response to oxidative stress. J. Biol. Chem..

[34] Szallasi Z., Denning M.F., Chang E.Y., Rivera J., Yuspa S.H., Lehel C., Olah Z., Anderson W.B., Blumberg P.M. (1995). Development of a rapid approach to identification of tyrosine phosphorylation sites: Application to PKC delta phosphorylated upon activation of the high affinity receptor for IgE in rat basophilic leukemia cells. Biochem. Biophys. Res. Commun..

[35] Ziegler W.H., Parekh D.B., Le Good J.A., Whelan R.D., Kelly J.J., Frech M., Hemmings B.A., Parker P.J. (1999). Rapamycin-sensitive phosphorylation of PKC on a carboxy-terminal site by an atypical PKC complex. Curr. Biol..

[36] Altman A., Kong K.F. (2016). Protein kinase C enzymes in the hematopoietic and immune systems. Annu. Rev. Immunol..

[37] Platten M., Eitel K., Wischhusen J., Dichgans J., Weller M. (2003). Involvement of protein kinase Cdelta and extracellular signal-regulated kinase-2 in the suppression of microglial inducible nitric oxide synthase expression by N-[3,4-dimethoxycinnamoyl]-anthranilic acid (tranilast). Biochem. Pharmacol..

[38] Baig M.S., Liu D., Muthu K., Roy A., Saqib U., Naim A., Faisal S.M., Srivastava M., Saluja R. (2017). Heterotrimeric complex of p38 MAPK, PKCδ, and TIRAP is required for AP1 mediated inflammatory response. Int. Immunopharmacol..

[39] Kim D.C., Kim S.H., Jeong M.W., Baek N.I., Kim K.T. (2005). Effect of rottlerin, a PKC-delta inhibitor, on TLR-4-dependent activation of murine microglia. Biochem. Biophys. Res. Commun..

[40] Tiwari R.L., Singh V., Singh A., Barthwal M.K. (2011). IL-1R-associated kinase-1 mediates protein kinase Cδ-induced IL-1β production in monocytes. J. Immunol..

[41] Kontny E., Kurowska M., Szczepańska K., Maśliński W. (2000). Rottlerin, a PKC isozyme-selective inhibitor, affects signaling events and cytokine production in human monocytes. J. Leukoc. Biol..

[42] Loegering D.J., Lennartz M.R. (2011). Protein kinase C and toll-like receptor signaling. Enzyme Res..

[43] Feng W., Song Y., Chen C., Lu Z.Z., Zhang Y. (2010). Stimulation of adenosine A(2B) receptors induces interleukin-6 secretion in cardiac fibroblasts via the PKC-delta-P38 signalling pathway. Br. J. Pharmacol..

