# Supplementary material for: Determination of the Role and Active Sites of PKC-Delta-Like from Lamprey in Innate Immunity
Source: Int J Mol Sci. 2019 Jul 9;20(13):3357. doi: 10.3390/ijms20133357 (PMC6650827; doi:10.3390/ijms20133357)
Supplement: Supplementary file 1 [file ijms-20-03357-s001.pdf]

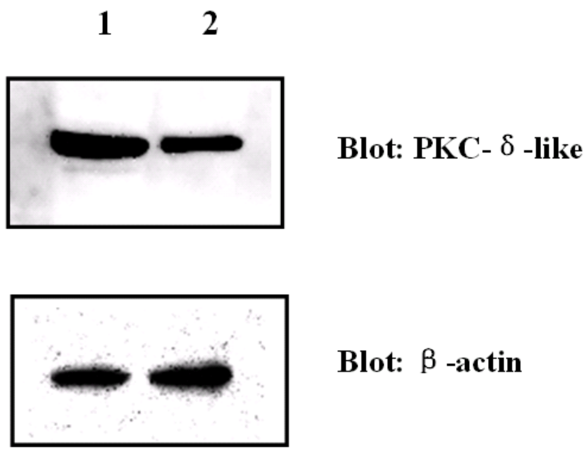

Figure S1 Expression of PKC-δ-like in the supraneural body and leukocytes of *L. japonica*. Lane 1 is the cell lysate of the supraneural body; lane 2 is the cell lysate of the leukocytes.

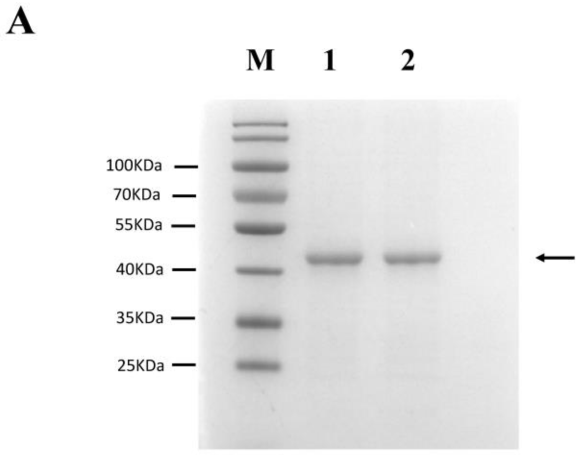

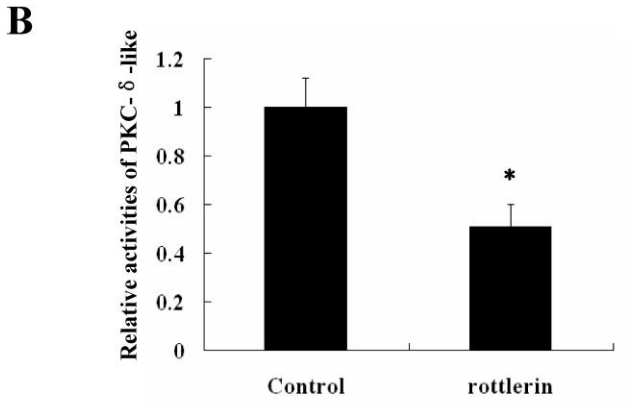

Figure S2 Activity assay of PKC-δ-like-CF treated without or with rottlerin in vitro. (A) Expression and purification of PKC-δ-like-CF recombinant protein. The recombinant proteins were expressed in *E. coli BL21* and analyzed through SDS-PAGE. M, protein marker; lane 1 and lane 2, purified PKC-δ-like-CF recombinant protein. (B) Activity assay of PKC-δ-like-CF treated without or with rottlerin (10 μmol/L). The asterisk indicates $p<0.05$.

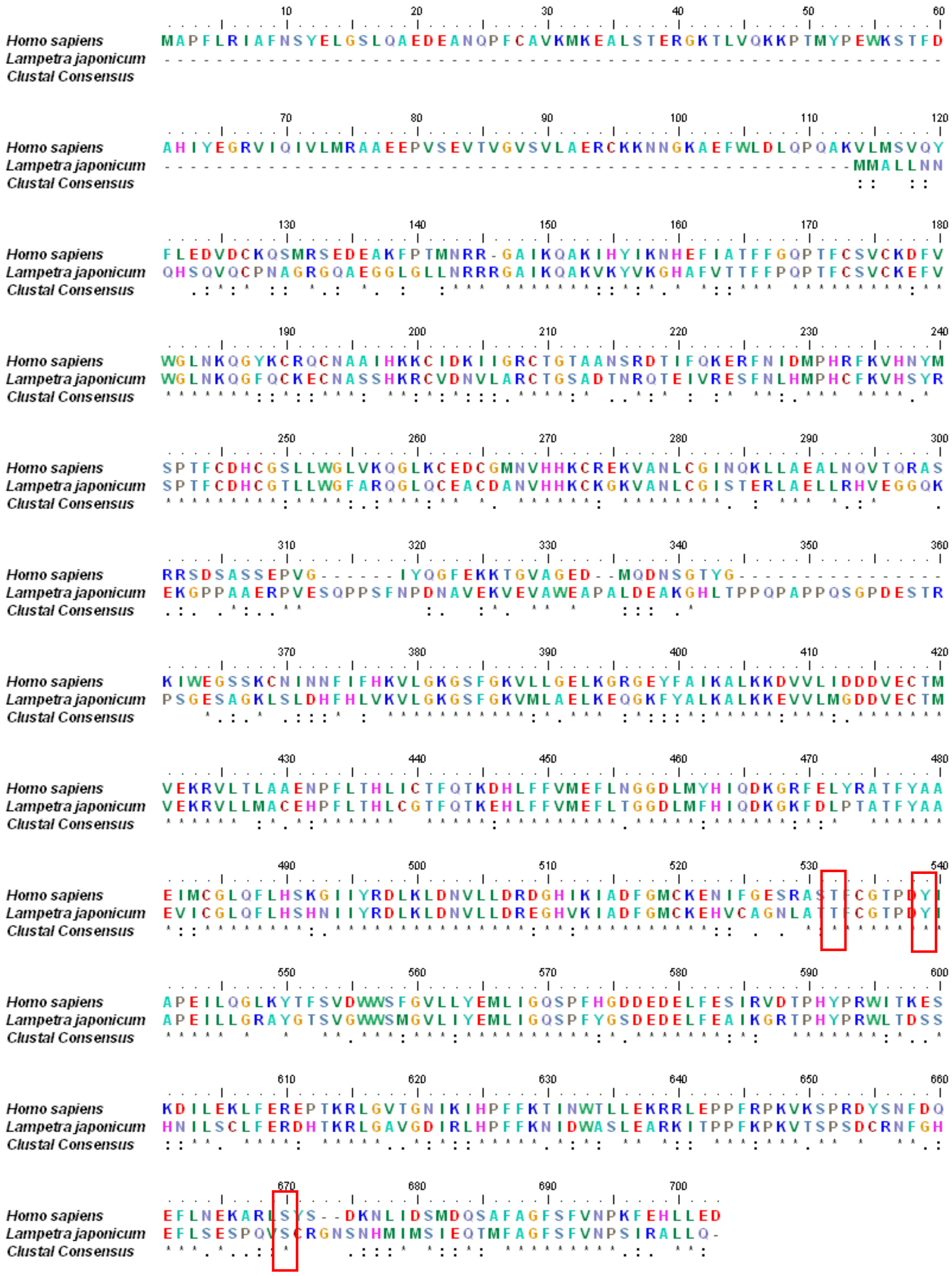

Figure S3 **S**equence alignment of PKC-δ-like with human PKC-δ. The following accession numbers of the amino acid sequences were extracted from the NCBI protein database: *Homo sapiens*: NP_006245; *Lampetra japonicum*: KX943554. The red boxes marked the active sites.
